# Recent Progress of Multifunctional Molecular Probes for Triple-Negative Breast Cancer Theranostics

**DOI:** 10.3390/pharmaceutics16060803

**Published:** 2024-06-14

**Authors:** Deyi Zhao, Zhe Li, Ding-Kun Ji, Qian Xia

**Affiliations:** 1 School of Life Sciences, Shanghai University, Shanghai 200444, China; zhao@shu.edu.cn (D.Z.); zhe-li@shu.edu.cn (Z.L.); 2 Institute of Molecular Medicine, Renji Hospital, Shanghai Jiao Tong University School of Medicine, Shanghai 200127, China; 3 Department of Nuclear Medicine, Institute of Clinical Nuclear Medicine, Renji Hospital, School of Medicine, Shanghai Jiao Tong University, Shanghai 200127, China

**Keywords:** drug delivery, triple-negative breast cancer, molecular probes, molecular imaging, treatment strategies

## Abstract

Breast cancer (BC) poses a significant threat to women’s health, with triple-negative breast cancer (TNBC) representing one of the most challenging and aggressive subtypes due to the lack of estrogen receptor (ER), progesterone receptor (PR), and human epidermal growth factor receptor 2 (HER2) expression. Traditional TNBC treatments often encounter issues such as low drug efficiency, limited tumor enrichment, and substantial side effects. Therefore, it is crucial to explore novel diagnostic and treatment systems for TNBC. Multifunctional molecular probes (MMPs), which integrate target recognition as well as diagnostic and therapeutic functions, introduce advanced molecular tools for TNBC theranostics. Using an MMP system, molecular drugs can be precisely delivered to the tumor site through a targeted ligand. Real-time dynamic monitoring of drug release achieved using imaging technology allows for the evaluation of drug enrichment at the tumor site. This approach enables accurate drug release, thereby improving the therapeutic effect. Therefore, this review summarizes the recent advancements in MMPs for TNBC theranostics, encompassing the design and synthesis of MMPs as well as their applications in the field of TNBC theranostics.

## 1. Introduction

Breast cancer (BC) is the most prevalent malignancy affecting women globally, constituting approximately 31% of new cases. In 2023, its mortality rate was alarmingly high at 15% [1]. The heterogeneity of BC is evident, leading to clinical categorization into three primary subtypes based on the hormone receptor (ER and PR) and HER2 (ERBB2) status: luminal ER-positive and PR-positive, further subdivided into luminal A and B; HER2-positive; and TNBC [2]. Among these subtypes, TNBC accounts for 10–15% of BC cases and carries the most unfavorable prognosis [3].

Common clinical treatments for TNBC include surgery, radiotherapy, and chemotherapy. However, these traditional treatments have limitations for the treatment of TNBC. Chemotherapy serves as the primary systemic medical treatment for TNBC, but TNBC patients exhibit a less favorable outcome after chemotherapy compared with patients with other BC subtypes [4,5]. Systemic chemotherapy, although a mainstay, often elicits poor responses and severe side effects, and leads to multiple drug resistance [6,7]. Beyond chemotherapy, immunotherapy has demonstrated effectiveness in various tumors and holds promise as a treatment strategy for TNBC. TNBC is particularly suitable for immunotherapeutic approaches due to factors such as tumor immune infiltration, the presence of neoantigens resulting from a mutational burden and higher genomic instability, and elevated levels of immune markers such as programmed death-ligand 1 (PD-L1) and programmed cell death protein-1 (PD-1). These markers are closely associated with tumor response, relapse, and overall outcomes [8]. However, TNBC’s high degree of heterogeneity, with multiple subtypes exhibiting distinct molecular profiles [9], poses a challenge as targeted therapies are lacking for these specific subtypes [10]. Consequently, there is an urgent need to develop effective new diagnostic and treatment methods and molecular tools for TNBC.

Currently, the field has seen a promising shift in the diagnosis and treatment of TNBC with the advent of new molecular diagnostic probes. MMPs that integrate target recognition as well as diagnostic and therapeutic functions introduce advanced molecular tools for TNBC theranostics. MMPs enable simultaneous drug delivery and visualization of the lesion with minimal off-target toxicity [11]. Benefiting from the targeting groups, MMPs can specifically bind to overexpressed receptors on the TNBC cell surface, thus highly improving the positive drug delivery [12]. By combining them with new therapy approaches such as photodynamic therapy (PDT) and photothermal therapy (PTT), MMPs may overcome multidrug resistance to highly improve the treatment efficiency of TNBC [13]. The introduction of advanced imaging technologies such as magnetic resonance imaging (MRI), fluorescence imaging, and positron emission tomography/single photon emission computed tomography (PET/SPECT) imaging endows MMPs with superior spatiotemporal imaging performance for TNBC diagnosis and visual precision treatment.

**Figure 1:**
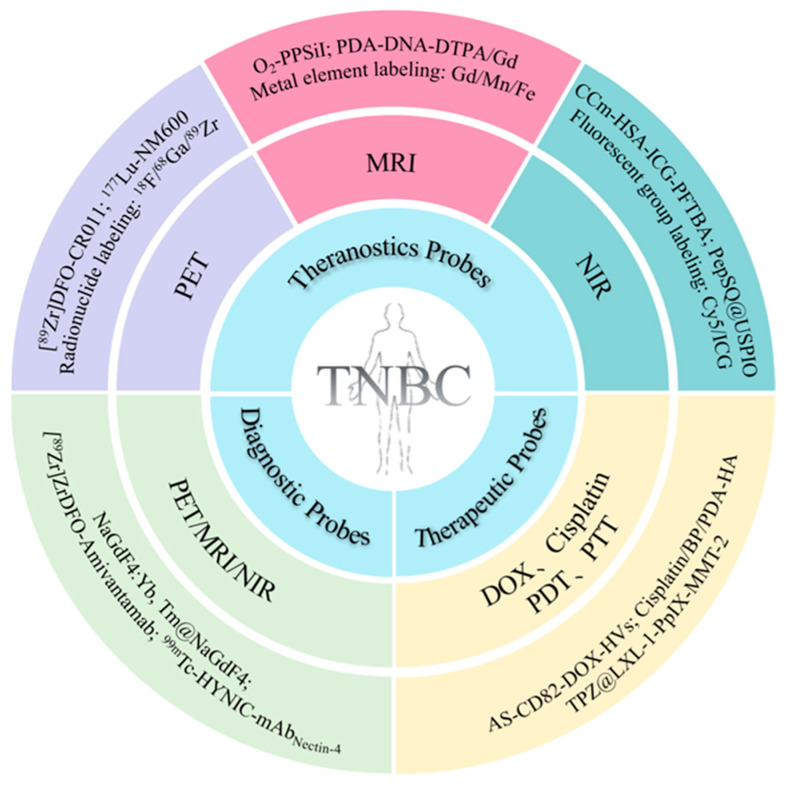
Schematic illustration of diagnostic, therapeutic, and theranostic probes for TNBC.

In this review, we summarize the recent advancements in MMPs for TNBC theranostics (Figure 1). Firstly, we introduce the basic principles and design strategies of MMPs. Subsequently, we discuss their specific applications in the early diagnosis and treatment, pathological evaluation, and prognosis assessment of TNBC in detail. Finally, we address the risks and challenges they encounter and provide insights into the future development direction and clinical application prospects of MMPs. This review aims to comprehensively summarize the application of MMPs in the diagnosis and treatment of TNBC, offering inspiration for further research and clinical practice to advance the precise treatment of this condition.

## 2. Design of Multifunctional Molecular Probes

**Figure 2:**
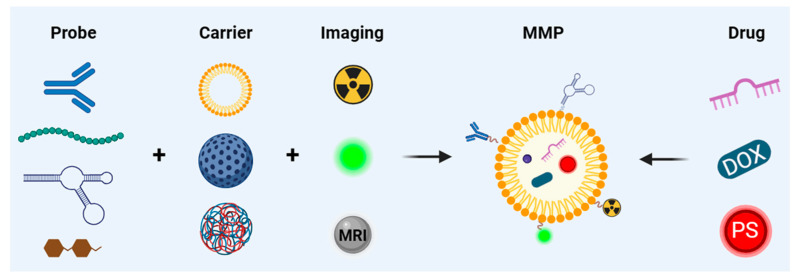
Schematic design of multifunctional molecular probes.

Multifunctional molecular probes integrate diverse capabilities within a single system, encompassing specific targeting, diagnosis, and therapy. When constructing molecular diagnostic probes for TNBC, it is crucial to carefully select specific targeting ligands, suitable carriers, and appropriate imaging methods (Figure 2).

### 2.1. Targeting Groups for Multifunctional Molecular Probes

In the design of MMPs for TNBC, it is crucial to select the appropriate targeting group. This involves considering the specificity, affinity, and biocompatibility of the molecule to ensure that it can accurately interact with TNBC-related molecules and exhibits stability and safety in vivo. Common ligands include peptides, aptamers, antibodies, and carbohydrates. They specifically bind to their receptors through ligand–receptor interactions to achieve targeted effects on TNBC cells, thus playing an important role in the diagnosis, treatment, and monitoring of TNBC [14].

Monoclonal antibody (mAb) is a burgeoning category of targeted cancer-treatment drugs, distinguished by a high specificity, extended serum half-life, robust affinity, and potent immune response function [15]. Monoclonal antibodies can achieve highly specific targeting by accurately recognizing and binding to specific antigens or receptors on the cell surface, thus exerting an excellent targeting ability in MMPs. Currently, several antibodies have been successfully applied to the construction of MMPs, including m276 [16], ICAM1 [17], and mAb_Nectin-4_ [18]. A combination of atezolizumab and nab-paclitaxel extended the progression-free survival of an intention-to-treat population and a PD-L1-positive subgroup in patients with metastatic TNBC [19]. This ingenious approach, utilizing antibodies for targeted drug delivery, augments drug enrichment at the tumor site, mitigating damage to normal cells and thereby enhancing the therapeutic efficacy. Guo et al. [17] designed an antibody–drug conjugate by coupling the ICAM1 antibody with monomethyl auristatin E (MMAE), verifying its excellent efficacy and safety as an effective antibody–drug conjugate candidate drug for TNBC treatment. However, it is crucial to acknowledge that antibodies carry certain limitations such as their substantial molecular weight and immunogenicity. Addressing these shortcomings necessitates ongoing optimization and improvement efforts [20].

Peptides have a series of advantages such as a simple structure, low synthesis cost, easy engineering [21,22], strong binding affinity, and high stability [23], which can efficiently reach the target and have great potential in the treatment of TNBC. To date, many peptides have been successfully applied to the construction of MMPs, including TH19P01, AXT050, RHFZD7, and PZ-128 [24]. For example, the TH19P01 peptide displayed high affinity to the sortilin (SORT1) receptor, which is highly expressed in TNBC [25]. To treat TNBC, AXT050, a collagen-IV-derived peptide with high affinity to tumor-associated integrin, was incorporated into MMPs to achieve antitumor and antiangiogenic effects [26]. Doxorubicin (DOX) is bound by peptide 18-4, targeting the TNBC surface-specific receptor keratin 1 (k1), and thus has specific toxicity for TNBC [27]. Based on the fact that integrins and NRP-1 are highly expressed in TNBC, the iRGD peptide, functioning as a tumor-homing and penetrating peptide, exhibits properties of binding to neurociliin-1 and integrin αvβ3, along with being internalized into TNBC cells. The use of radionuclides to label the iRGD peptide enables the in vivo imaging of TNBC [28]. However, peptides also have some shortcomings such as being unstable and easily decomposed in vivo and having a short half-life [29], Further modifications may be needed to improve their functions.

An aptamer is one type of single-stranded DNA or RNA. It possesses the remarkable ability to fold into a three-dimensional structure, enabling it to selectively bind to specific target molecules [30]. It can be screened from a random library using a technique called systematic evolution of ligands by exponential enrichment (SELEX). Leveraging its high specificity and molecular targeting prowess, aptamers have emerged as an ideal tool to detect cancer surface markers and monitor treatment. To date, many aptamers have been successfully applied to the construction of MMPs, including AS1411 [31,32,33,34,35], aptamer (S1.5) [36], LXL 1 [37], CL4 aptamer [38], PD-L1 aptamer [39], sTN 145 aptamer [40], and anti-EGFR aptamer [41]. Highly efficient and nuclease-resistant aptamers targeting platelet-derived growth factor receptor β (PDGFR β) offer promising prospects to inhibit TNBC growth. These aptamers not only contribute to antitumor immunity but also augment the responsiveness of anti-PD-L1 antibodies in the context of TNBC growth and the formation of lung metastases [42]. Epidermal growth factor receptor (EGFR) is overexpressed in about 60% of TNBC and is associated with a poor prognosis. When the cl4 aptamer was injected into xenograft mice, it resulted in the inhibition of the formation of an integrin αvβ3–EGFR complex, thereby inhibiting tumor growth [43]. AS1411 can specifically target the overexpressed nucleolar receptor on the surface of TNBC cells. It achieves its therapeutic effect by competing with bcl-2 mRNA for nucleolin binding, thereby destabilizing bcl-2 in MDA-MB-231 [44]. Kang et al. [35] constructed AS1411 aptamer-modified PEG@PLGA nanoparticles (NPs) encapsulated by perfluorohexane (PFH) and the anticancer drug DOX. A further synergistic treatment of TNBC was achieved by the introduction of aptamers to enhance tumor-targeted imaging and improve drug target enrichment. Notably, their status as a pure nucleic-acid chain renders them biocompatible and non-immunogenic; however, their vulnerability to nuclease degradation is an aspect worth considering [45,46].

Carbohydrates, constituting the third class of informational biomolecules following proteins and nucleic acids, play pivotal roles in both physiological and pathological events. These encompass critical functions in cell signaling, differentiation, proliferation, tumor metastasis, inflammatory response, and viral infection [47]. Notably, carbohydrates excel at achieving the precise targeting of cells by specifically binding to sugar receptors on the surface of tumor cells. To date, many sugar ligands have been developed for the construction of MMPs, including hyaluronic acid (HA) [48,49,50,51] and chitosan oligosaccharide [52,53]. HA is a naturally occurring glycosaminoglycan found in the body’s connective tissue. It can target the CD44 receptor, an integral membrane glycoprotein overexpressed on several tumor-cell surfaces, including TNBC. Chitosan oligosaccharide can also target CD44 to deliver MMPs to TNBC cancer cells. Dong et al. [54] modified liposomes using HA. This modification helped to deliver DOX and epoprostenol (EPS) to tumor cells in vivo via active targeting, which together inhibited tumor growth and metastasis in TNBC. Ding et al. [52] designed a chitosan-encapsulated liposome to encapsulate a photosensitizer (HPPH) and a hypoxia-activated prodrug (TH302) into a hydrophobic bilayer. The modification of chitosan allowed for better tumor targeting and the resulting liposome could be used to aid the diagnosis of TNBC by fluorescent imaging and generate antitumor therapy through synergistic PDT and chemotherapy. Leveraging these sugar molecules, drugs, or fluorescent compounds can be conjugated to prepare MMPs, capitalizing on the sugar transport mechanisms inherent in TNBC cells. This innovative strategy enhances drug targeting, minimizes toxicity to normal cells, and presents a promising avenue for therapeutic advancement.

### 2.2. Carriers of Multifunctional Molecular Probes

Nanocarriers play a pivotal role in the construction of MMPs. Advanced nanocarriers for MMP construction have been explored, including liposomes, hydrogels, micelles, dendrimers, polymer NPs, and DNA nanostructures [14]. These carriers not only shield drugs from degradation during transport but also amplify drug enrichment at the disease site. Furthermore, they allow control over pharmacokinetic and pharmacodynamic distribution, thereby mitigating adverse reactions [55].

Organic nanocarriers, including NPs such as liposomes, micelles, dendritic polymers, and polymer nanocarriers, offer a less toxic and versatile platform for MMP construction. These nanocarriers can be tailored to enhance drug bioavailability by improving solubility and facilitating transport across biological membranes, thereby increasing drug absorption [56]. Dai et al. [57] targeted overexpressing integrin-α3 in TNBC models with cyclic octapeptide LXY (Cys-Asp-Gly-Phe (3,5-DiF)-Gly-Hyp-Asn-Cys) attached to liposomes carrying a dual drug, i.e., DOX and rapamycin. This dual drug targeted approach resulted in improved efficacy compared with a free drug. Poly (acrylic acid)-g-PEG, i.e., PAA-g-PEG, copolymeric micelles carrying DOX were developed by Sun et al. [58] for an efficient reduction in lung metastasis and 4T1 mouse breast-tumor growth. The effective delivery of therapeutic agents such as DOX, paclitaxel, and miRNA to the tumor site with the help of nanocarriers achieves an efficient loading and release of the drugs as well as increasing the enrichment and utilization of the drugs at the lesion site [59].

Inorganic nanocarriers, including materials such as gold, magnetic nanocarriers, quantum dots, and mesoporous silica, have been utilized in the construction of MMPs. Among these inorganic nanocarriers, mesoporous silica nanoparticles (MSNs) stand out as a novel drug-carrier platform with advantageous biological properties, including biocompatibility, biodegradability, and non-toxicity. Additionally, MSNs feature distinct structural attributes such as tailored mesoporous channels, a uniform pore-size distribution, and a high surface area, enhancing their effectiveness as drug-delivery systems [60,61]. He et al. [62] designed a silica nanosystem (SNS) with Nano-Ag as the core and a complex of MnO_2_ and DOX as the surrounding mesoporous silica shell. This system was coated with anti-PD-L1 to target the PD-L1 receptor, which is highly expressed on the surface of tumor cells. An SNS appears to have favorable biosafety and antitumor effects, and may be a novel therapy for the treatment of TNBC.

### 2.3. Imaging Modalities for Multifunctional Molecular Probes

As the prognosis of TNBC is worse than other BC subtypes, early detection, diagnosis, and treatment are essential methods to improve its prognosis. A diagnosis of TBNC primarily relies on morphological changes to reflect lesion characteristics. Many imaging technologies have been successfully developed, including X-ray photography, ultrasound, CT, MRI, and PET-CT imaging techniques [63]. The introduction of imaging groups to MMPs endows them with the ability to detect TNBC. Additionally, as integral components of molecular diagnostic probes, imaging techniques can guide therapeutic MMPs to tumors or facilitate external triggers to induce cargo release, achieving a more precise treatment for TNBC. 

MRI is a commonly used medical imaging technology due to its high resolution and non-invasiveness. Many MRI molecules have been explored to construct MMPs, including As/Mn-NHs [64] and PDA-DNA-DTPA/Gd [65]. Through the labeling of drugs with magnetic NPs or MRI contrast agents, the distribution of drugs becomes observable in MRI, enabling a localization and distribution assessment of the drugs. Zhai et al. [64] investigated self-activated As/Mn-NHs by co-biomineralizing arsenic manganite within hollow albumin nanocages. This innovative approach demonstrated smart high-contrast MRI detection and synergistic arseno-therapeutic effects on TNBC tumor models. 

Radionuclides have gained increasing importance in modern medicine, playing a crucial role in both the imaging and treatment of primary and metastatic tumors. Based on their functions, they are typically categorized into two types. Diagnostic radionuclides are primarily used for tracer diagnosis in patients and include isotopes such as ^99m^Tc, ^68^Ga, ^18^F, and ^13^N [66]. On the other hand, therapeutic radionuclides necessitate substantial accumulation in specific organs and rapid blood clearance. The accumulation in specific organs should be significantly higher than that in normal tissues. Examples of therapeutic radionuclides include ^131^I, ^177^Lu, and ^32^P [67]. To date, many radioactive molecules have been explored to construct MMPs, including ^99m^Tc-HYNIC-iRGD [28], ^99m^Tc-HYNIC-mAb_Nectin-4_ [18], and ^89^Zr@CS-GA-MLPs [68]. Huang et al. [69] synthesized diZD, a high-affinity vascular endothelial growth factor receptor (VEGFR)-targeting agent. This agent was further labeled with ^177^Lu and ^64^Cu using a 1,4,7,10-tetraazacyclododecane-1,4,7,10-tetraacetic acid (DOTA) chelating agent, resulting in the therapeutic agent ^177^Lu-DOTA-diZD and PET imaging agent ^64^Cu-DOTA-diZD. Micro-PET/CT imaging demonstrated a specific accumulation of ^64^Cu-DOTA-diZD in 4T1 mouse tumors, while therapeutic ^177^Lu-DOTA-diZD exhibited remarkable antitumor efficacy.

Optical imaging uses light and the special properties of photons to obtain detailed images of organs, tissues, cells, and even molecules. In comparison to other molecular imaging modalities such as MRI, ultrasound imaging, and PET-CT, it boasts high spatiotemporal resolution and sensitivity with minimal invasiveness [70,71]. Near-infrared (NIR) fluorophores in particular exhibit enhanced optical properties compared with traditional fluorescent dyes. Operating within a specific window (λ = 700–1000 nm), NIR dyes offer improved utility owing to the deeper penetration depth of the excitation light, high spatial precision of detection, and minimized background interference of the emission signal [72,73]. Many NIR fluorescent molecules have been explored to construct MMPs, including Cy7 [38] and IR780 [74]. Zhang et al. [74] established a biomimetic nanoplatform using a hybrid membrane derived from white blood cells and platelets (LPHMs) combined with dendritic macroporous mesoporous silica nanoparticles (DLMSNs). Within the macropores of the DLMSNs, the NIR fluorescent dye IR780 and the chemotherapeutic drug DOX were co-loaded, resulting in DLMSN@DOX/IR780 (DDI) NPs. The biodistribution and tumor accumulation of NPs were evaluated by observing the fluorescence of IR780. Upon NIR laser irradiation, the LPHM@DDI NPs exhibited synergistic cytotoxicity and apoptosis-inducing activity in TNBC cells. 

## 3. Application of Multifunctional Molecular Probes in TNBC

TNBC, as one of the most aggressive and worst prognostic subtypes of breast cancer, faces a number of diagnostic and therapeutic challenges due to a lack of surface receptors. With the advancement of nanotechnology, biomedical science is increasingly focused on the development of contrast agents and drug-delivery carriers for a more accurate and targeted co-delivery of diagnostic and therapeutic drugs in cancer treatment [14]. MMPs have been designed to encapsulate drugs within nanocarriers, linking specific ligands and imaging moieties for the diagnosis and treatment of TNBC (Table 1).

### 3.1. MMPs for the Targeted Diagnosis of TNBC

Currently, challenges such as inaccurate diagnosis using non-specific contrast agents, false-positive results, and the influence of examiner experience remain limiting and decisive factors in validating TNBC diagnoses [14]. The evolving field of nanotechnology offers promise, particularly through the development of nanocarrier-based molecular diagnostic probes, which have the potential to address and overcome these limitations.

To increase the sensitivity of detection, some radionuclide-based MMPs have been developed with high affinity to TNBCs. Recent studies have highlighted nectin cell adhesion molecule 4 (Nectin-4), also known as poliomyelitis virus receptor-related protein 4, which is expressed in 62% of TNBC cases and is associated with a poor prognosis [85]. In the study by Shao et al. [18], the authors utilized a mAb against Nectin-4 as a carrier to design a radio probe, ^99m^Tc-HYNIC-mAb_Nectin-4_. This radio probe exhibited significant Nectin-4-specific targeting both in vitro and in vivo. Micro-SPECT/CT images revealed that tumor radioactivity in the experimental group could be identified after 3 h, gradually increasing over time. Notably, significant radioactivity was observed at the tumor site, with a favorable target-background contrast at 24 and 36 h. In the study by Cavaliere et al. [77], a radiolabeled bispecific antibody called [^89^Zr]ZrDFO-Amivantamab was successfully prepared and evaluated for its pharmacological and imaging properties. The researchers compared the imaging quality of [^89^Zr]ZrDFO-Amivantamab with a radiolabeled antibody iso-type control, [^89^Zr]ZrDFO-IgG1. They evaluated the performance of both antibodies in terms of their pharmacological and imaging properties. PET imaging demonstrated an increased tumor accumulation of [^89^Zr]ZrDFO-Amivantamab in MDA-MB-468 and MDA-MB-231 compared with [^89^Zr]ZrDFO-IgG1, resulting in an excellent imaging contrast.

**Figure 3:**
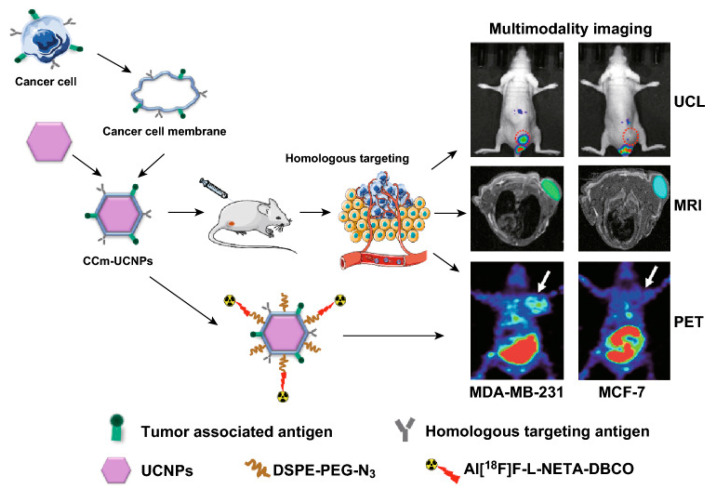
Illustration of the cancer-cell membrane-coated Gd^3+^-doped upconversion nanoparticles (CCm-UCNPs) used to differentiate between MDA-MB-231 and MCF-7 tumor-bearing mice models by homologous-targeting multimodality imaging, including UCL, MRI, and PET. Colors/red dotted circles/white arrows indicate tumor site. Reprinted from [76].

Multimodal imaging or multiplexed imaging refers to the simultaneous production of signals for more than one imaging technique. Each imaging modality has its inherent advantages and disadvantages. Therefore, in order to obtain multidimensional biological information, different imaging modalities can be rationally combined to realize ultrasensitive in vivo multimodal precise imaging of TNBCs. MMPs with various imaging capabilities show great application potential in the targeted diagnosis of TNBC. Upconversion nanoparticles (UCNPs) stand out as superior luminescent substances. This superiority arises from the facile combination of rare-earth ions with paramagnetic ions such as Gd^3+^, enabling the simultaneous visualization of tumors through upconversion luminescence (UCL) [86] and MRI [75,87]. In a study by Fang et al. [76], the authors designed a probe using the cancer-cell membrane of MDA-MB-231 to modify Gd^3+^-doped UCNPs NaGdF_4_:Yb,Tm@NaGdF_4_ (CCm_231_-UCNPs) (Figure 3). This probe showcased homologous-targeting and immune-escaping abilities. Harnessing the UCL of UCNPs, the paramagnetism of Gd^3+^, and click chemistry enabled a surface modification to label ^18^F. CCm-UCNPs were employed for ultrasensitive in vivo UCL/MRI/PET multimodal precise imaging of TNBC. The multiple imaging modalities used together confirmed that the tumor sites in the MDA-MB-231 group showed much higher uptake of the probe than the MCF-7 group, thus the NPs had the efficacy to distinguish between the MDA-MB-231 and MCF-7 hormonal mouse models in vivo. This may be a potential method to achieve the integration of diagnosis and treatment as well as to monitor and evaluate therapeutic effects.

### 3.2. MMPs for the Targeted Therapy of TNBC

**Figure 4:**
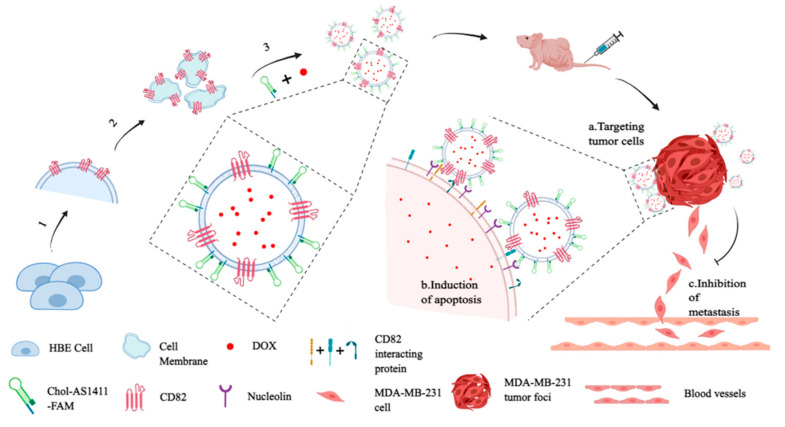
Generation of “triple-punch” strategy cell membrane-derived nanovesicles follows three steps: 1. overexpression of the metastasis-suppressive molecule CD82 in HBE cells; 2. extraction of cell membrane fragments and preparation of cell membrane vesicles; and 3. modification with aptamers and loading of DOX into membrane vesicles. After intravenous (i.v.) administration, AS-CD82-DOX-HVs can specifically bind to TNBC by AS1411 targeting (a), effectively delivering DOX to induce cancer-cell apoptosis (b) and providing metastasis inhibition from CD82 overexpression (c), which can enhance antitumor efficacy [34]. Copyright 2024, American Chemical Society.

As one of the most malignant subtypes of BC, TNBC lacks effective therapeutic strategies and has a poor prognosis. Currently, the therapeutic options for TNBC are still limited to surgery, adjuvant chemotherapy, and radiotherapy [8]. MMPs provide a new direction for the precision treatment of TNBC. Zhang et al. [34] generated an exosome-mimetic nanovesicle system integrated with CD82 overexpression, AS1411 conjugation, and DOX delivery (Figure 4). Exosomes are an efficient drug-delivery system and CD82 is an exosome-enriched tumor metastasis inhibitory molecule. Nucleic-acid aptamer AS1411 specifically targets TNBC cells with a high expression of nucleolin. CD82 enrichment effectively inhibits TNBC cell migration. DOX loading effectively inhibits TNBC cell proliferation and induces apoptosis. The results showed that the MMPs significantly inhibited liver metastasis and ameliorated the level of apoptosis in cancer cells.

MMPs for the controlled delivery of drugs such as cisplatin, which can be combined with other therapeutic modalities for synergistic antitumor effects and metastasis inhibition, are highly desirable for the treatment of TNBC. Li et al. [50] reported a multifunctional molecular probe based on black phosphorus (BP) nanosheets, which were composed of cisplatin, BP, polydopamine (PDA), and HA for the control of cisplatin delivery and the inhibition of tumor growth. In this study, the BP nanosheets were sequentially modified with PDA and HA. This molecular probe enabled the targeted and on-demand delivery of cisplatin to inhibit tumorigenesis and metastasis through a synergistic chemotherapy/photothermal mode, providing a method for BP-based antitumor therapy. 

To improve the efficacy of TNBC treatment, Zhang et al. [37] developed a novel strategy combining bioreductive therapy with photodynamic PDT. Thin-shell hollow mesoporous Ia3d silica NPs (named MMT-2) were functionalized with protoporphyrin IX (PpIX) and a DNA aptamer (LXL-1) and loaded with tirapazamine (TPZ), resulting in the formation of TPZ@LXL-1-PpIX-MMT-2 nanocarriers, which were designed to target the human BC cells, MDA-MB-231. The PpIX molecules were utilized to react and consume oxygen at the tumor site to produce cytotoxic reactive oxygen species (ROS). Low oxygen levels in the microenvironment activated the bioreductive prodrug TPZ into toxic radicals, which not only scavenged hypoxic tumor cells but also enhanced the efficacy of PDT. The results showed that this multifunctional molecular probe facilitated in vitro and in vivo targeting and significantly reduced the tumor volume in xenograft mouse models. A histological analysis also showed that this nanocarrier was effective at killing tumor cells in hypoxic regions.

### 3.3. MMPs for the Theranostics of TNBC

Compared with independent treatment methods and imaging modes, therapeutic MMPs have the advantage of integrating imaging and treatment into the same platform, thereby achieving the synchronous diagnosis and treatment of tumors. Additionally, they can monitor treatment effectiveness, enhancing the overall efficacy of tumor treatment. MRI, fluorescence imaging, and PET have successfully been integrated into MMPs to detect microscopic lesions, track drug release, and evaluate the effectiveness of TNBC treatment.

#### 3.3.1. Theranostic Probes Based on MRI

MRI holds significant promise for advances in the visualized drug delivery of TNBC. Through its capability to monitor drug distribution, release kinetics, treatment response, and drug targeting, MRI enables the optimization of treatment plans and enhances overall treatment effectiveness. Sun et al. [65] developed a therapeutic diagnostic MRI nanoprobe (PDA-DNA-DTPA/Gd) for the imaging and treatment of a 4T1 tumor-bearing mouse model. A PDA-DNA-DTPA/Gd solution was injected into the tumor site of 4T1 tumor-bearing mice and whole-body MRI scans were conducted at various time points. An optimal contrast of the MRI signal at the tumor site was observed after 30 min. Upon irradiating the tumor site with an 808 nm laser, the non-irradiated tumor area remained unchanged and the contrast of the tumor area after laser irradiation significantly decreased over time. After 1 h, the MRI signal of the tumor site was significantly lower than that of non-laser irradiation. An intertumoral injection revealed that the antitumor effect of laser-irradiated PDA-DNA-DTPA/Gd-DOX in vivo was significantly higher than that of other treatments, leading to an improved survival rate of mice. This study further demonstrated the feasibility of synergistic PTT and chemotherapy, enhancing the therapeutic efficacy of the nanoprobe.

**Figure 5:**
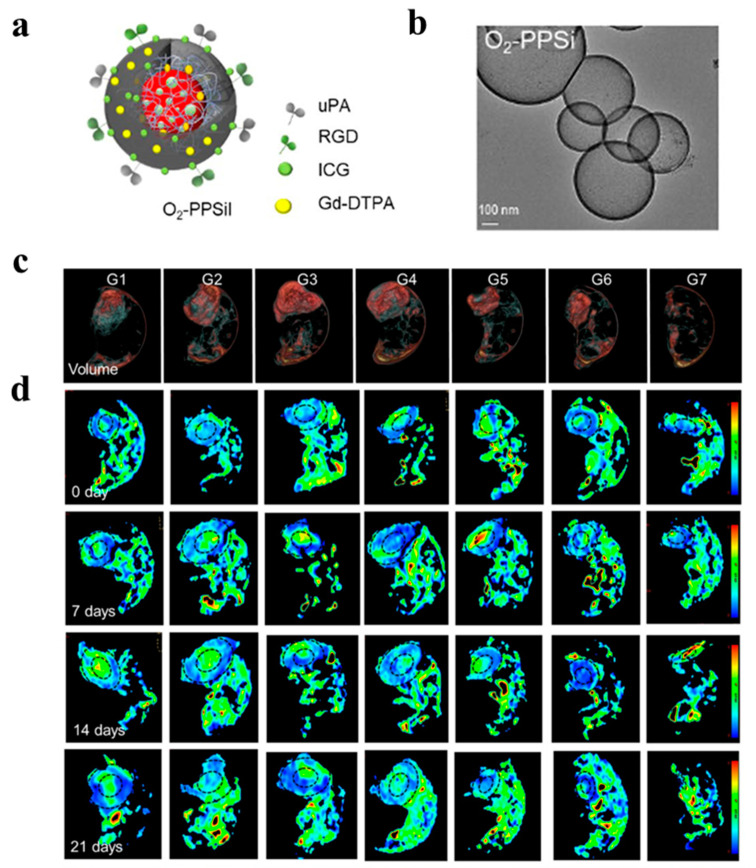
The structural characterization of O_2_-PPSiI and in vivo antitumor therapeutic efficacy for NIR-triggered O_2_-PPSiI. (**a**) The structure of O_2_-PPSiI. (**b**) TEM image of O_2_-PPSi. (**c**) The representative images for tumor volume derived from 3D-CUBE T2WI in each group 21 days after the treatment. (**d**) IVIM-DWI-derived mapping of tumors in each group before and after the treatment. Black circles indicate the tumor site. (G1: Saline; G2: Laser; G3: PTX; G4: O_2_-PPSiI; G5: PPSiI + Laser; G6: O_2_-PSiI + Laser; G7: O_2_-PPSiI + Laser) Reprinted from [79].

In another study, Zhang et al. [78] introduced a NIR-responsive on-demand oxygen-releasing nanoplatform (O_2_-PPSiI) guided by MRI, with the perspective that alleviating the hypoxic microenvironment of TNBC could enhance the antitumor efficacy (Figure 5). The nanoplatform was administered into the tail vein of mice with MDA-MB-231 orthotopic tumors and exposed to an 808 nm NIR laser for 5 min. The antitumor activity of O_2_-PPSil was assessed using structural and functional MRI. O_2_-PPSiI accumulation at the tumor site peaked 8 h after the tail-vein injection. A further finding was that the synergistic chemophototherapy induced by the improvement in tumor hypoxia under NIR laser irradiation effectively suppressed tumor-cell proliferation. The nanosystem achieved a precise O_2_ release through the NIR-responsive rupture of silica shell. The real-time, dynamic biodistribution of O_2_-PPSiI was quantitatively analyzed using sensitive MRI of the tumor. This multifunctional molecular probe, which enabled the real-time monitoring of the nanosystem delivery, enhanced the anti-TNBC efficacy of chemotherapy by alleviating the hypoxic microenvironment while inhibiting its invasion and metastasis.

#### 3.3.2. Theranostic Probes Based on NIR Fluorescence Imaging

**Figure 6:**
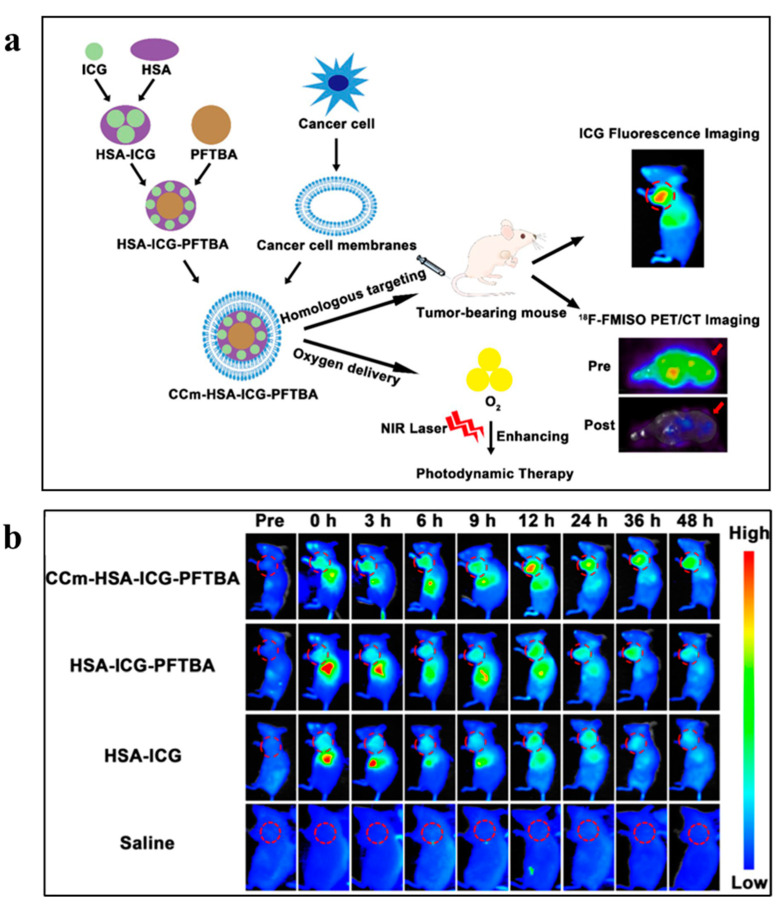
(**a**) Illustration of a biomimetic oxygen-delivery nanoprobe. Red arrows indicate tumor site. (**b**) In vivo fluorescence images of TNBC xenografts after the injection of CCm-HSA-ICG-PFTBA, HSA-ICG-PFTBA, HSA-ICG, and saline at different time points. Red circles indicate the tumor site. Reprinted from [80].

In vivo NIR fluorescence imaging is an emerging medical imaging method, with applications in both basic experimental and clinical research. Fluorescence, known for its rapid response and high sensitivity, faces challenges such as the inherent fluorescence of tissues and scattering, which have constrained its development. Consequently, insufficient diagnosis and treatment options for TNBC have prompted research into TNBC-therapy probes, aiming to achieve imaging-guided treatment [87,88,89,90]. Fang et al.’s study (Figure 6) [79] focused on the development of a bionic oxygen-delivery nanoprobe, CCm-HSA-ICG-PFTBA, designed for homologous targeting and hypoxia relief at tumor sites to enhance the efficacy of TNBC xenotransplantation. The results from ICG fluorescence imaging revealed that the fluorescence in the tumor tissues of the CCm-HSA-ICG-PFTBA group was the most intense and persisted for 48 h. In contrast, the liver content was the lowest, indicating that the cancer-cell membrane coating reduced uptake in the reticuloendothelial system (RES). For in vivo PDT treatment, the tumor volume and weight of the CCm-HSA-ICG-PFTBA with NIR group were both smallest among all the groups and significantly decreased compared with the untreated group. This suggests that this nanoprobe has the potential to be clinically transformed into an effective oxygen-delivery agent to alleviate TNBC tumor hypoxia while enhancing the therapeutic efficacy of PDT. 

**Figure 7:**
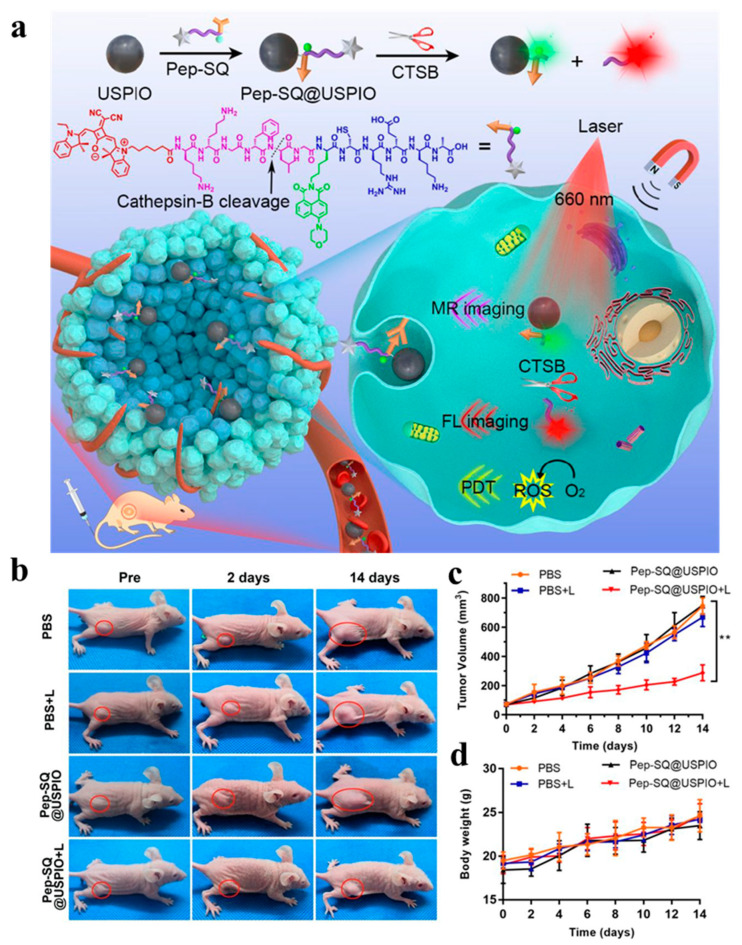
(**a**) Fibronectin targeting and CTSB-activatable Pep-SQ@USPIO nanoprobe for MR/fluorescence imaging and enhanced PDT of TNBC. (**b**) Photographs of MDA-MB-231 tumor-bearing mice during PDT. Red circles indicate the tumor site. (**c**) Tumor-volume curves of mice. Data present as mean ± SD, *n* = 5 (** *p* < 0.01) (**d**) Weight-growth curves of mice [81]. Copyright 2020, American Chemical Society.

Due to its unique tumor microenvironment that is distinct from other subtypes, TNBC exhibits a higher metastasis rate and a more aggressive nature. Recognizing these characteristics, numerous studies have focused on targeting various factors within the tumor microenvironment, including tumor-shedding gangliosides, lactic acid, and oncoproteins [91]. Fibronectin has emerged as a promising targeted marker for TNBC. In Wang et al.’s study (Figure 7) [80], a therapeutic nanoprobe named PepSQ@USPIO was designed to specifically target fibronectin, activated by endogenous cathepsin B. The study utilized magnetic resonance/near-infrared fluorescence (MR/NIRF) imaging for TNBC to guide PDT. The fluorescence signal was transferred to the MDA-MB-231 tumor site 2 h after a Pep-SQ@USPIO injection. The fluorescence intensity at the tumor site gradually increased from 2 to 6 h after the injection and robust tumor-specific fluorescence was observed at 6 h. The therapeutic aspects were compared with the controls and a decrease in tumor volume over time was observed in mice treated with Pep-SQ@USPIO and laser irradiation.

Benefiting from the low toxicity and safety of NPs and leveraging biodegradable NPs, poly (lactic-co-glycolic acid) (PLGA) was approved by the Food and Drug Administration (FDA) for clinical use. Agnello et al. [38] utilized PLGA as a carrier for NPs; this facilitated the connection of a novel EGFR aptamer, creating a system for the targeted delivery of cisplatin. To track the nanovectors’ biodistribution in vivo alongside tumor targeting, NIR Cy7 was covalently labeled onto the urokinase plasminogen activator receptor (uPAR) surface. Confocal microscopy and an in vivo imaging analysis of the TNBC xenografts demonstrated the prepared NPs’ targeting ability for EGFR-positive tumor cells. The effective encapsulation of cisplatin in targeted nanocarriers showcased a superior killing effect on tumor cells compared with bare drugs and nanocarriers without or with interference aptamers. The aptamer’s targeting and cisplatin’s chemotherapeutic effect on the DNA damage of cancer cells synergistically achieved the goal of targeted therapy. Additionally, the authors noted that this nanoparticle could overcome TNBC resistance to EGFR inhibitors.

#### 3.3.3. Theranostic Probes Based on PET

PET primarily employs radionuclides, wherein radioactive material generates positrons within the body. These positrons interact with other electrons, enabling comprehensive body scanning and imaging. The clinical significance of PET imaging has steadily increased, especially since the approval of ^18^F-2-fluoro-2-deoxy-D-glucose (^18^F-FDG). PET imaging not only furnishes quantitative molecular-level information but also elucidates underlying biological features. As a result, it finds frequent applications in tumor diagnosis, the staging of newly diagnosed malignant tumors, restaging patients post-radiotherapy, and monitoring treatment progress.

The heightened expression of glycoprotein non-metastatic B (gpNMB) in TNBC is correlated with recurrence and metastasis, making it a viable target for the antibody–drug conjugate glembatumumab vedotin (CDX-011). In a study led by Marquez-Nostra et al. [81], ^89^Zr-labeled glembatumumab ([^89^Zr]DFO-CR011) was employed to categorize patients based on their response to CDX-011. The findings indicated that [^89^Zr]DFO-CR011 holds promise as a potential pretherapeutic diagnostic tool for CDX-011, specifically in targeting gpNMB. This underscores its potential for clinical translation within the realm of radiopharmacology.

**Figure 8:**
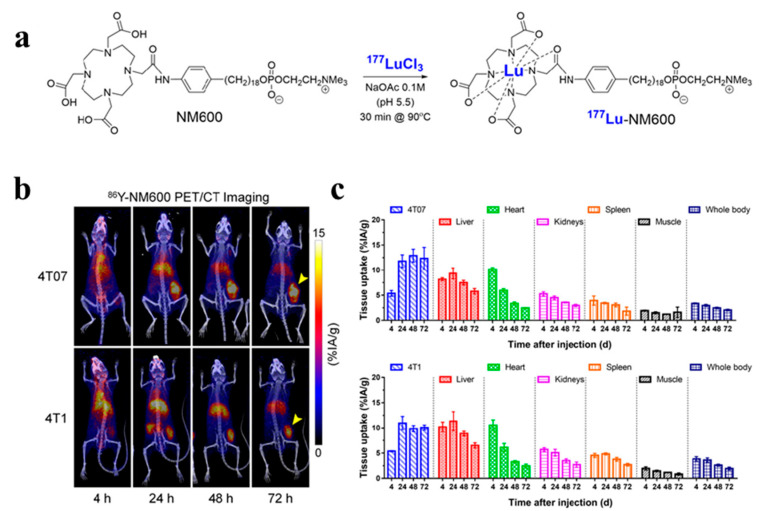
(**a**) Schematic representation of ^177^Lu radiolabeling of NM600. (**b**) ^86^Y-NM600 PET/CT imaging in murine models of TNBC. Yellow arrows indicate tumor site. (**c**) Results of quantitative region of interest analyses of PET imaging in BC mice [82]. Copyright 2020, Society of Nuclear Medicine and Molecular Imaging.

In Hernandez et al.’s [82] study (Figure 8), radiation therapy utilized ^177^Lu-labeled alkylphosphocholine (NM600) complemented with PET imaging and ^86^Y to monitor NM600 biodistribution. The ^177^Lu-NM600 treatment was well-tolerated, except for mild cytopenia. Notably, the high tumor uptake observed indicated the potential of ^177^Lu-NM600 to inhibit tumor growth and extend survival in patients lacking effective treatment options. Subsequent studies will explore the therapeutic potential of high linear energy transfer (LET) radionuclides such as ^225^Ac, ^227^Th, or ^212^Pb for metastatic TNBC.

Recently, advancements in granzyme B and downstream effector serine proteases of cytotoxic T cells have provided valuable biomarkers to predict the effectiveness of immunotherapy. In a study conducted by Napier et al. [83], it was discovered that using granzyme B for PET imaging ([^68^Ga]-NOTA-GZP) could offer early insights into immune checkpoint blockades in TNBC following chemotherapy. To the best of the authors’ knowledge, this was the first study utilizing [^68^Ga]-NOTA-GZP to evaluate the impact of immune checkpoint inhibition combined with chemotherapy on effector cell activation in a syngeneic orthotopic mouse TNBC model. The findings revealed that GZP-PET could offer quantitative information regarding effector cell activation in two types of TNBC tumor and also monitor the tumor volume. Furthermore, it could accurately differentiate the tumor response to treatment.

Maternal embryonic leucine zipper kinase (MELK) assumes a crucial role in regulating tumor-cell growth, particularly in TNBC where it is abundant, and has emerged as a promising target for molecular imaging and treatment. In the study led by Tang et al. [84], a high-affinity MELK inhibitor (OTSSP167) was employed for PET imaging and a molecular probe, ^11^C-methoxy-OTSSP167, was synthesized and evaluated for its application in TNBC PET imaging. The PET imaging results substantiated that the ^11^C-methoxy-OTSSP167 molecular probe could effectively distinguish between high and low MELK expressions. The study also anticipated future clinical use by proposing a shift to ^18^F due to its longer half-life compared with ^11^C. Additionally, the use of hydrophilic and non-toxic polyethylene glycol (PEG) was suggested to enhance water solubility, improve in vivo pharmacokinetics, and reduce liver absorption.

## 4. Conclusions and Outlook

As precision medical technology advances, MMPs have emerged as a novel and effective tool for TNBC. The integration of molecular imaging technology with molecular probes has given rise to a new class of diagnostic and therapeutic probes, presenting an innovative treatment strategy. This approach enables the targeted imaging of tumor sites, allowing the real-time monitoring of drug distribution and targeting effects in vivo. Consequently, it enhances the accuracy and efficacy of diagnosis, marking a significant advance in precision medicine for TNBC.

Although MMPs have rapidly developed in recent years, they are still in their infancy. Many challenges need to be addressed before they enter the clinic. Firstly, for now, a lack of highly specific biomarkers is the main reason hindering probe efficacy. Therefore, finding specific biomarkers for TNBC is crucial for the development of effective MMPs. Secondly, the biosafety of the nanocarrier is one of the biggest obstacles in the clinical transformation of MMPs. This issue could be resolved by the development of biodegradable or biocompatible carriers. Thirdly, simple, efficient, and accurate MMP construction methods are highly needed to increase their functionality and efficacy in TNBC.

In conclusion, MMPs exhibit vast potential in the diagnosis and treatment of TNBC. As technology advances and research deepens, there is a strong belief that MMPs will contribute to the formulation of more accurate and effective diagnosis and treatment strategies for patients with TNBC.

## Figures and Tables

**Table 1:** Summary of composition and application of MMPs.

MMPs	Targeting Group	Carriers	Imaging Group	Drug	Application	Cancer Type	Mouse Strain	Refs
AS-CD82-DOX-HVs	AS1411	HVs		DOX/CD82		MDA-MB-231	Balb/C-nude	[34]
TPZ@LXL-1-PpIX-MMT-2	LXL-1	MMT-2		PpIX		MDA-MB-231	Balb/C-nude	[37]
Cis-Pt@PNPs-CL4	CL4	PNPs	Cy7	Cisplatin	NIR	MDA-MB-231/BT-549	Balb/C-nude	[38]
Cisplatin/BP/PDA-HA	HA	BP		Cisplatin		4T1	Balb/C	[50]
As/Mn-NHs	EPR	Albumin	Mn^2+^	Arsenic trioxide (ATO)	MRI	4T1/CT26/HT29	Balb/C	[64]
PDA-DNA-DTPA/Gd	EPR	PDA	DTPA/Gd	PDA/DOX	MRI	4T1	Balb/C-nude	[65]
^177^Lu-DOTA-diZD/^64^Cu-DOTA-diZD	diZD		^64^Cu	^177^Lu	PET	4T1	Balb/C	[69]
LPHM@DDI NPs	LPHM	DLMSN	IR780	DOX	NIR	4T1	Balb/C	[74]
NaGdF_4_:Yb, Er nanocrystals,	EPR	NaYF4 nanocrystals	Gd^3+^		MRI/NIR	LS180	Balb/C-nude	[75]
cancer-cell membrane mimic Gd3+-doped upconversion nanoparticles (CCm-UCNPs)	Homologous targeting	Cancer-cell membrane	UCNPs		UCL/MRI/PET	MDA-MB-231/MCF-7	Balb/C-nude	[76]
^99m^Tc-HYNIC-mAb_Nectin-4_/mAbNectin-4-ICG	mAb_Nectin-4_		^99m^Tc/ICG		SPECT/NIR	MDA-MB-468	Balb/C-nude	[18]
[^89^Zr]ZrDFO-Amivantamab	Amivantamab		^89^Zr		PET	MDA-MB-468/MDA-MB-231/MDA-MB-453	Balb/C-nude	[77]
O_2_-PPSiI	RGD/uPA	PLGA	ICG/Gd-DTPA	PTX	NIR/MRI	MDA-MB-231	Balb/C-nude	[78]
CCm-HSA-ICG-PFTBA	Homologous targeting	Cancer-cell membrane	ICG/^18^F	PFTBA	NIR/PET	4T1	Balb/C-nude	[79]
PepSQ@USPIO	Cys-Arg-Glu-Lys-Ala (CREKA)	USPIO	SQ/USPIO	SQ	NIR/MRI	MDA-MB-231/MCF-7	Balb/C-nude	[80]
[^89^Zr]DFO-CR011	CR011		^89^Zr		PET	MDA-MB-157/MDA-MB-468/MDA-MB-231	Balb/C-nude	[81]
^177^Lu-NM600/^86^Y-NM600	NM600	NM600	^86^Y	^177^Lu	PET	4T1/4T07	Balb/C-nude	[82]
[^68^Ga]-NOTA-GZP	GZP		^68^Ga	PTX/anti-PD-1/anti-CTLA4	PET	4T1/E0771	Balb/C/C57Bl6	[83]
^11^C-methoxy-OTSSP167	OTSSP167		^11^C		PET	MCF-7/MDA-MB-231	Balb/C-nude	[84]

## Data Availability

No new data were created or analyzed in this study.

## Abbreviations

BC	Breast cancer
BP	Black phosphorus
DFO	Desferrioxamine
DOX	Doxorubicin
EGFR	Epidermal growth factor receptor
EPR	Enhanced permeability and retention
EPS	Epalrestat
ER	Estrogen receptor
FDA	Food and Drug Administration
gpNMB	Glycoprotein non-metastatic B
HA	Hyaluronic acid
HER2	Human epidermal growth factor receptor 2
ICAM1	Intercellular adhesion molecule-1
IHC	Immunohistochemistry
LET	Linear energy transfer
mAb	Monoclonal antibody
MELK	Maternal embryonic leucine zipper kinase
MMAE	Monomethyl auristatin E
MMP	Multifunctional molecular probe
MR	Magnetic resonance
MRI	Magnetic resonance imaging
Nectin-4	Nectin cell adhesion molecule 4
NIR	Near-infrared
NIRF	Near-infrared fluorescence
NPs	Nanoparticles
PD-1	Programmed cell death protein-1
PD-L1	Programmed death-ligand 1
PDA	Polydopamine
PDT	Photodynamic therapy
PEG	Polyethylene glycol
PET	Positron emission tomography
PFH	Perfluorohexane
PLGA	Poly (lactic-co-glycolic acid)
PpIX	Protoporphyrin IX
PR	Progesterone receptor
PTT	Photothermal therapy
PTX	Paclitaxel
RES	Reticuloendothelial system
ROS	Reactive oxygen species
SPECT	Single photon emission computed tomography
TNBC	Triple-negative breast cancer
TPZ	Tirapazamine
UCL	Upconversion luminescence
UCNPs	Upconversion nanoparticles
uPA	Urokinase plasminogen activator
uPAR	Urokinase plasminogen activator receptor
